# The protective effect of angiotensin II type I receptor blocker (valsartan) on behavioral impairment, NLRP3, BDNF, and oxidative stress in the brain tissue of ovariectomized female rats

**DOI:** 10.14814/phy2.70003

**Published:** 2024-10-23

**Authors:** Salih Erdem, Hale Sayan Özaçmak, İnci Turan, Meryem Ergenç

**Affiliations:** ^1^ Ahmet Erdoğan Vocational School of Health Services, Pathology Program Zonguldak Bülent Ecevit University Zonguldak Turkey; ^2^ Department of Physiology, Faculty of Medicine Zonguldak Bülent Ecevit University Zonguldak Turkey; ^3^ Ahmet Erdoğan Vocational School of Health Services, Anesthesia Program Zonguldak Bülent Ecevit University Zonguldak Turkey

**Keywords:** BDNF, brain oxidative stress, depression and anxiety‐like behavior, IL‐1β, NLRP3

## Abstract

Depression and anxiety are common mental health disorders affecting thoughts, behaviors, and emotions. This study aimed to investigate the effect of the angiotensin II type I receptor blocker (AT1RB), valsartan, on menopause‐induced depression and anxiety‐like behaviors, and to elucidate possible mechanisms of action by measuring levels of nod‐like receptor protein 3 (NLRP3), interleukin‐1beta (IL‐1β), brain‐derived neurotrophic factor (BDNF), and oxidative stress in brain tissue. Thirty‐two Wistar albino female rats were randomly divided into four groups (*n* = 8 per group): Control, AT1RB, OVX, and AT1RB + OVX. Following the bilateral ovariectomy (OVX) protocol, physiological saline was used as valsartan solvent, in a maximum volume of 0.4 mL, and valsartan was administered via intragastric gavage at a dose of 40 mg/kg/day. Depression and anxiety‐like behaviors were assessed using the forced swimming test and open field test. Levels of oxidative stress markers, NLRP3, IL‐1β, BDNF, and CREB were analyzed in the hippocampus and prefrontal cortex tissues. Behavioral tests indicated that depression and anxiety‐like behaviors significantly increased in OVX rats (*p* < 0.01), while AT1RB treatment significantly reduced these behaviors (*p* < 0.05). In the hippocampus of OVX rats, oxidative stress (*p* < 0.01), NLRP3 (*p* < 0.05), and IL‐1β (*p* < 0.01) levels were elevated, whereas BDNF levels were significantly decreased (*p* < 0.01). AT1RB treatment significantly improved oxidative stress parameters (*p* < 0.05) and BDNF levels (*p* < 0.01) but did not significantly affect the increased levels of NLRP3 and IL‐1β in OVX rats. In conclusion, AT1RB has a therapeutic effect on menopause‐induced depression and anxiety‐like behaviors, likely by reducing oxidative stress and increasing BDNF production in the hippocampus.

## Graphical abstract

In female Wistar albino rats, an experimental menopause model was induced with a bilateral overoectomy protocol. Forced swimming and open field behavioral tests were applied to analyze the effects of the experimental menopause model and valsartan treatment on depression and anxiety‐like behaviors. At the end of the experimental procedure, MDA, GSH, BDNF, IL‐1B, and NLRP3 levels were analyzed in the hippocampus to analyze the physiopathological mechanism of menopause‐related depression and the therapeutic effects of valsartan on these mechanisms.

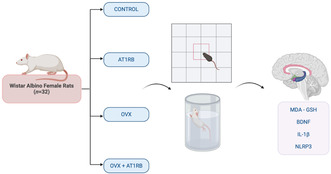

## 1 INTRODUCTION

Menopause is a natural process that marks the end of the menstrual cycle and fertility due to a depletion of follicles. Menopause can happen earlier in life due to factors such as surgical procedures, chemotherapy, radiotherapy, and premature ovarian failure (Averkova & Yakushevskaya, 2022). Hormone replacement therapy is the primary option to prevent or reduce menopause‐related symptoms. Estradiol hormone application is an effective method for treating menopause‐induced depression. However, complementary studies have reported that long‐term hormone treatments are associated with an increased risk of stroke and breast cancer (Hyun et al., 2022). Experimental animal studies use ovariectomized (OVX) rats, which are considered the classical model to test new treatments for pathophysiological events that occur with menopause (Cipriani et al., 2022; Kim et al., 2020; Lan et al., 2020).

Depression and anxiety are common mental health disorders affecting thoughts, behaviors, and emotions. Women are twice as likely as men to experience depression (Kessler et al., 2003). Menopause‐induced depression is a well‐known symptom of the menopausal period, with symptoms similar to major depressive disorders. Research indicates that during the menopausal transition, some women may be at a higher risk of developing depression compared to their premenopausal years, with an increased risk even for those who have never experienced depression before (Soares, 2007). Studies have reported that the incidence rate of depression is higher in menopausal women, ranging from 5.9% to 23.8% (Zeng et al., 2019). Women in the perimenopausal or early postmenopausal stages are two to four times more likely to experience a major depressive episode (Bromberger et al., 2011). Additionally, the prevalence of depressive symptoms and suicidal ideation tends to increase with advancing menopausal stages, including pre‐menopause (An et al., 2022). The mechanisms of depression are controversial, but monoamine neurotransmitters are the most widely accepted. However, their inability to explain some clinical findings has shifted the research focus to new alternative depression mechanisms (Duman & Monteggia, 2006; Hamon & Blier, 2013; Marshe et al., 2017; Moeller et al., 2014). The neurotrophin family comprises four structurally related proteins with similar functions, namely nerve growth factor (NGF), brain‐derived neurotrophic factor (BDNF), neurotrophin‐3 (NT‐3), and neurotrophin‐4 (NT‐4). BDNF plays an important role in regulating various types of neurons, including sensory neurons, retinal ganglion cells, spinal motor neurons, certain cholinergic neurons, and some dopaminergic neurons. The synthesis of BDNF is known to be influenced by neuronal activity in the brain and plays a vital role in synaptic transmission and plasticity (Jun et al., 2022; Xiyang et al., 2020). The production of BDNF is reduced in neurodegenerative diseases, including Alzheimer's, Parkinson's, and Huntington's diseases, according to post‐mortem studies (Manoharan et al., 2016). BDNF concentration decreases in individuals with major depressive disorder and increases after antidepressant drug treatment (Shimizu et al., 2003). Inflammation and oxidative damage are critical factors in the pathogenesis of depression and brain diseases, similar to other pathological conditions. Reducing or blocking neuroinflammation appears to be an effective approach to delaying neurodegenerative diseases. Several studies have reported that circulating inflammatory cytokines, such as interleukin (IL)‐1β, IL‐6, and tumor necrosis factor (TNF‐α), increase in patients with mood and anxiety‐related disorders (Clerici et al., 2009). Among inflammatory cytokines, IL‐1β release plays a central role in regulating neuroinflammation by activating astrocytic Nod‐like receptor protein (NLRP), a multi‐protein complex of the innate immune system. NLRP3 is a potential therapeutic target in a mouse model of chronic mild stress‐induced depression, and it may be a central mediator in the treatment of lipopolysaccharide (LPS)‐induced depression (Iwata et al., 2013; Zhang et al., 2014, 2015). The renin‐angiotensin system (RAS) plays a crucial role in inflammation and is associated with various brain functions such as learning, memory, and mood. Angiotensin II (Ang II), which plays a major role in the RAS, plays a role in regulating sympathetic, neuroendocrine, stress responses, cerebral blood flow, and brain inflammatory response. Most of the effects of Ang II occur through stimulation of the Ang II type I receptor (AT1R), and the overstimulation of AT1R in the brain is associated with neuroinflammation, microglial activation, oxidative stress, and neuronal loss. Previous studies have shown that inhibiting angiotensin‐converting enzyme (ACE) or blocking AT1R by angiotensin II type I receptor blocker (AT1RB) can ameliorate microglial activation, cytokine production, oxidative stress, apoptosis, neuronal loss, and the effects of inflammation on behavior (Abareshi et al., 2016; Benicky et al., 2011; Sun et al., 2015; Villapol et al., 2015; Xu, Xu, et al., 2015). In addition, AT1RBs appear to be more effective than ACE inhibitors in neuroinflammation (Bhat et al., 2016). While the underlying mechanisms of menopause‐related depression are poorly understood and effective treatments are limited, current research implicates a complex interplay of neurotransmitter dysregulation, cytokines, oxidative stress, hypothalamic–pituitary–adrenal (HPA) axis dysfunction, and neurotrophic factor alterations for the pathophysiology of menopause‐related depression. Understanding these underlying mechanisms is crucial to developing targeted therapeutic interventions and improving outcomes for individuals affected by depression. The beneficial effects of AT1RB on brain oxidative damage and neuroinflammation suggest that it is a novel and alternative method for treating menopause‐induced depression and anxiety disorders. The objective of this research is to explore the impact of AT1RB therapy on depression and anxiety‐like behaviors triggered by menopause, as well as the levels of NLRP3, IL‐1β, BDNF, and oxidative stress in the hippocampal and prefrontal cortex tissues.

## 2 MATERIALS AND METHODS

### 2.1 Experimental animals and groups

All surgical procedures applied in this experiment followed the terms of the Zonguldak Bülent Ecevit University Animal Experiments Local Ethics Committee (protocol number: 2018‐08‐05/04). This study was designed by ARRIVE guidelines 2.0.

**FIGURE 1:**
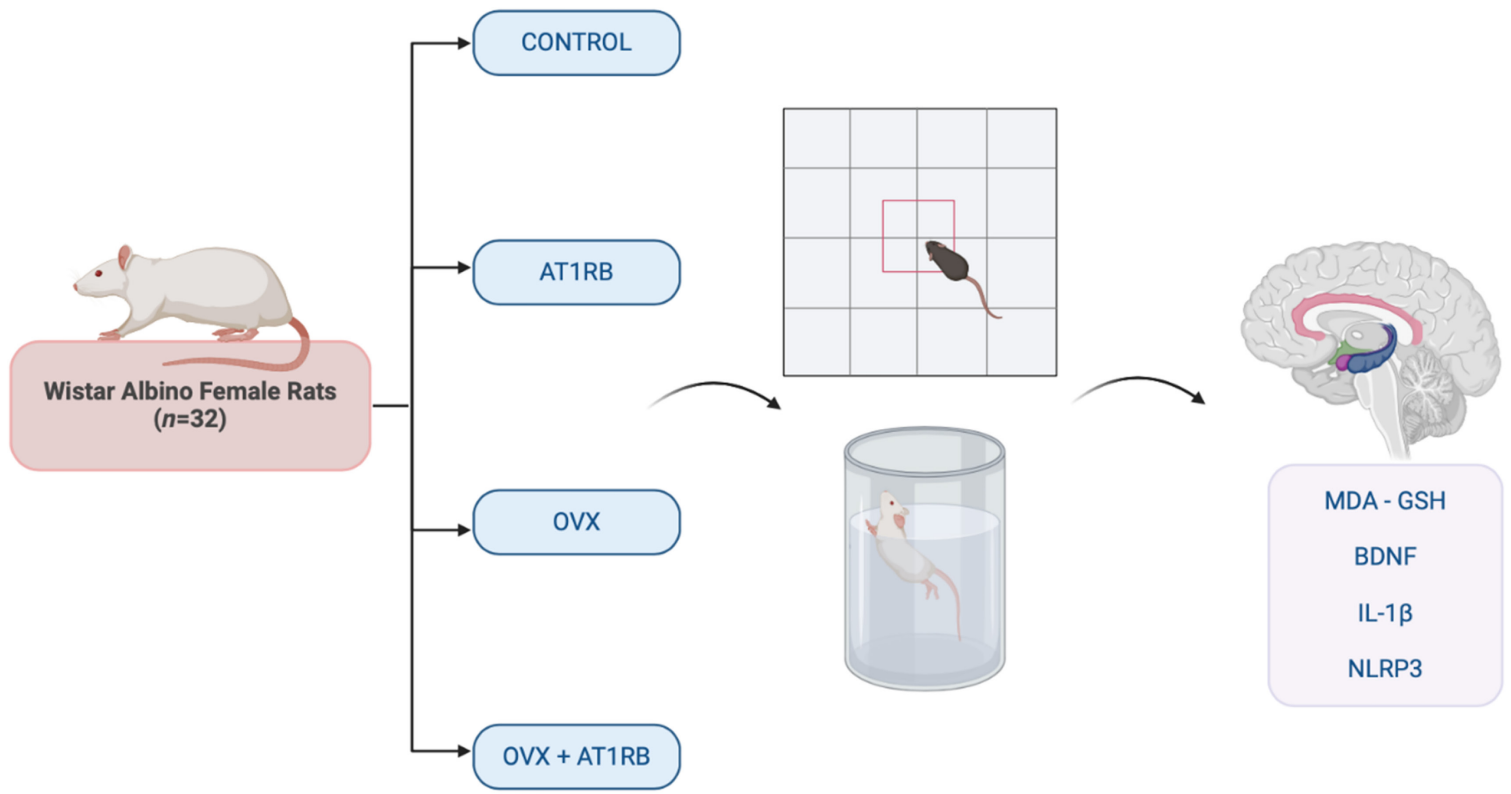
Graphical summary of the experimental procedure. In female Wistar albino rats, an experimental menopause model was induced with a bilateral overoectomy protocol. Forced swimming and open field behavioral tests were applied to analyze the effects of the experimental menopause model and valsartan treatment on depression and anxiety‐like behaviors. At the end of the experimental procedure, MDA, GSH, BDNF, IL‐1β, and NLRP3 levels were analyzed in the hippocampus to analyze the physiopathological mechanism of menopause‐related depression and the therapeutic effects of valsartan on these mechanisms.

In this research, a total of 32 female Wistar albino rats were utilized. These rats were between the ages of 7–8 months and weighed between 200 and 220 grams. The rats were sourced from Zonguldak Bülent Ecevit University Experimental Animal Production Laboratory and were housed in suitable conditions within the same laboratory. They were given standard pellet feed (Rat Feed, DSA Agrifood Products Inc., Turkey) and had access to tap water at all times. The rats were kept at a constant temperature of 21 ± 2°C, with a humidity level of 65%, and a day/night schedule of 12 h each. The subjects were randomly selected and formed into four equal groups. Control; Surgical and gavage stress was created. Ovariectomy and drug administration were not performed. AT1RB; Surgical stress was created and 40 mg/kg/day of valsartan was given orally. OVX; The ovaries of the experimental animals were removed bilaterally. OVX+ AT1RB; Ovariectomy was performed and 40 mg/kg/day valsartan treatment was administered (Figure 1).

### 2.2 Ovariectomy protocol

Female rats were randomly divided into groups in Eurostandard Type‐IV cages before starting the experimental protocol. OVX and OVX+ AT1RB subjects were anesthetized with ketamine (90 mg/kg) + xylazine (10 mg/kg) intraperitoneally. The lateral dorsum of female rats was shaved and cleaned with batikon. A small incision (1 cm) was made on the back skin and the right and left ovaries were ligated distally with sterile silk suture, and then the ovaries were dissected. The incised skin was stitched with sterile silk suture, cleaned with batikon and placed in cages. The same incision was applied to Control and AT1RB subjects and then closed again. All surgical procedures were performed under aseptic conditions (Da Silva et al., 2016; Jöhren & Saavedra, 1996).

### 2.3 Drug and treatment

After 16 days of surgical operation, rats from AT1RB and OVX + AT1RB were given valsartan, an AT1RB, at a dose of 40 mg/kg/day for 14 days. The drug was administered at the same time every day to all rats. On the other hand, control and OVX rats were administered with a maximum of 0.4 mL of physiological serum, which is a drug solvent, via intragastric gavage (Wilkinson‐Berka et al., 2007).

#### 2.3.1 Operational description

Depression and Anxiety‐Like Behaviors: Forced Swimming Test, it was used to measure the helplessness behavior of rats. Time spent sedentary during the test was considered a sign of depression. Open Field Test, it was used to evaluate the anxiety‐like behavior of rats. Latency at center, rearing, and number of frames passed were used to determine anxiety level.

In‐vivo blood pressure measurement: Before the subjects were sacrificed, a cannula was placed in the right carotid artery under anesthesia and the cannula was connected to the pressure transducer. Blood pressures (mmHg) were monitored and recorded using the BioPack system.

Hippocampus and Prefrontal Cortex Sampling: NLRP3, IL‐1β, BDNF and CREB levels were measured by ELISA method using tissue samples taken from these regions.

Oxidative Stress Parameters: Malondialdehyde (MDA) and Glutathione (GSH) Levels: Used as markers of oxidative stress. MDA and GSH levels were measured by spectrophotometric methods.

### 2.4 Behavioral tests

#### 2.4.1 Forced swimming test

The Porsolt test, also referred to as the forced swimming test, is a widely utilized method to assess the efficacy of antidepressants. This test involves placing an experimental animal, usually a rat, in a cylindrical glass bowl filled with water, measuring 30 cm in height and maintained at a temperature of 22 ± 1°C. The rat's escape behavior and periods of immobility are recorded while its swimming movements are observed with a Logitech C920, WebCam for subsequent analysis. The immobility periods are considered an indication of depression‐like behavior (Abelaira et al., 2013; Czéh et al., 2016; Hu et al., 2020). Before the test, all groups undergo pre‐training by swimming for 15 min on the first day. After 24 h, the water in the bowl is replaced, and the actual test is conducted. The rat is observed for 5 min, and its movements are recorded by the camera for later independent analysis.

#### 2.4.2 Open field test

The open field test was developed by Wash and Cummins in 1976 and is a simple and useful test used to measure anxiety‐like behavioral parameters of experimental animals in behavioral studies. The number of lines crossed, rearing, number of central square entries, and time spent in the central square are used to measure locomotor activity and anxiety parameters. The frequency of these behaviors indicates increased locomotor activity and a low level of anxiety‐like behavior (Ramos et al., 1997; Steimer, 2011). The open field test was carried out in a plexiglass box divided into equal squares of 80 × 80 × 30 cm. The experimental animal was left in the center square and its movements were recorded for 5 min (Logitech C920, WebCam). After each subject, the open field apparatus was cleaned with 20% alcohol. Behavioral parameters such as rising onto the limb (rearing), the number of frames passed, and the time until the animal moved after being released to the center (latency) were evaluated by independent researchers through video recordings.

### 2.5 Hemodynamic analysis

Subjects in all groups were anesthetized before sacrifice. For mean arterial blood pressure measurement, the right carotid artery was cannulated with a polyethylene tube containing heparin (100 IU/mL), and the mean arterial blood pressure was measured for 10 min and recorded to analyze the results (Blood pressure transducer, SS 13 L, Biopac Systems, California, USA).

### 2.6 Spectrophotometric measurement

Immediately after blood pressure measurement, the rats were sacrificed by applying high‐dose anesthesia (ketamine + xylazine) intravenously. The hippocampus and prefrontal cortex were dissected. The hippocampus and prefrontal cortex tissues of the rats, which were kept at −80°C, were removed and allowed to thaw on ice. 10% trichloroacetic acid (TCA, Sigma Chemical Co., St. USA) was added nine times the weighed tissue weight and all tissues were homogenized with a homogenizer until an equal homogeneous solution was formed.

#### 2.6.1 Malondialdehyde analysis

Malondialdehyde analysis (MDA) determination was performed according to the method of Casini et al. (1986). The prepared homogenates were centrifuged at 20oC/3000 *g*/15 min. 1% butylhydroxy toluene (BHT, Sigma Chemical Co., St. USA) and 0.67% thiobarbituric acid (TBA, Sigma Chemical Co., St. Louis, MO, USA) were added to the supernatant. The samples were boiled for 15 min and read spectrophotometrically at a wavelength of 535 nm.

#### 2.6.2 Reduced glutathione (GSH) analysis

GSH levels were studied according to the method of Aykac et al. (1985). The supernatant was centrifuged in microcentrifuge tubes at 20°C/3000 *g*/8 min. 1 mL 0.3 M Na_2_HPO_4_ (Sigma Chemical Co., St. USA) and 125 μL dithiobisnitrobenzoate (Sigma Chemical Co., St. USA) were added to the sample supernatant. Samples were read spectrophotometrically at a wavelength of 412 nm.

### 2.7 Enzyme‐linked immune sorbent assay

Brain tissues of rats at −80°C were prepared with a mechanical homogenizer in phosphate buffer solution (pH: 7.4). Homogenates were centrifuged at 4°C/3000 *g*/20 min and used in supernatant analyses. Supernatants were analyzed by enzyme‐linked immune sorbent assay (ELISA) method for BDNF (Cloud‐clone Corp., SEA011Ra, USA), IL‐1β (Cloud‐clone Corp., SEA563Ra,USA), NLRP3 (Cloud‐clone Corp., SEK115Ra, USA) according to commercial kits. The results were read according to the curves in each catalog (Awareness, Chromate 4300, USA).

### 2.8 Biostatistical analysis

Statistical evaluation was made using SPSS 19.0 (SPSS Inc., Chicago, IL, USA). Descriptive statistics are expressed as mean ± standard deviation. Kruskal–Wallis analysis of variance was used to compare the four groups. In the Kruskal–Wallis analysis of variance, a pairwise comparison of subgroups was made with the Dunn test. The relationship between two numerical variables was examined with Spearman correlation analysis, and a *p*‐value of <0.05 was considered significant for all evaluations.

## 3 RESULTS

### 3.1 ATR1B effect on mean arterial blood pressure

The comparison between the weights (*p* = 0.734) of the experimental animals and the mean arterial blood pressure (MABP) values (*p* = 0.736) measured after 30 days of drug administration was not found to be statistically significant. Statistical results show that valsartan treatment and the experimental menopause model did not affect weight gain and mean arterial blood pressures in the subjects.

### 3.2 AT1RB reduced depression and anxiety‐like behavior İncreased by Ovariectomy

**FIGURE 2:**
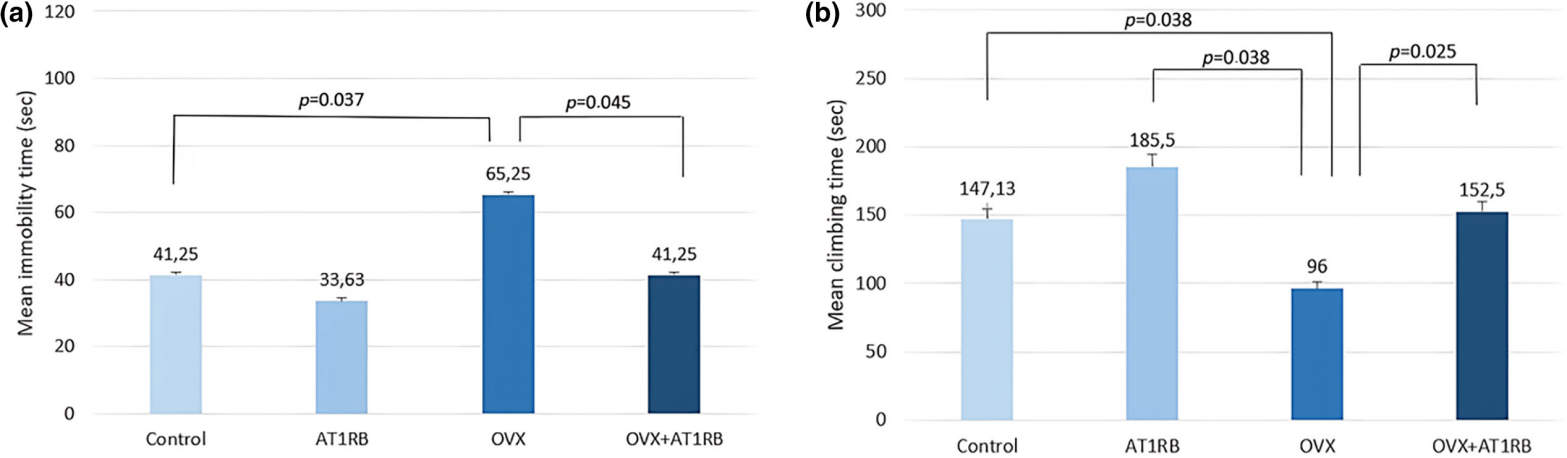
(a, b) Comparison of immobility and climbing times in the forced swimming test. Ovariectomy increased helplessness and depression‐like behavior. Valsartan treatment prevented neurobehavioral disorders. Intergroup comparisons were analyzed with the Kruskal–Wallis test, and pairwise comparisons were analyzed with the post‐hoc Dunn test. Data are given as mean ± standard deviation.

**FIGURE 3:**
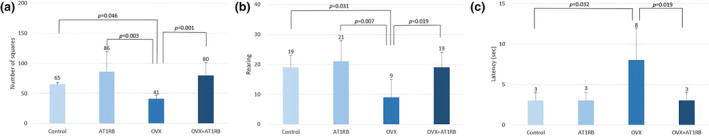
(a–c): Open field test number of squares and rearings, comparison of latency time. Ovariectomy increased anxiety‐like behavior. Valsartan treatment prevented anxiety‐like behaviors. Intergroup comparisons were analyzed with the Kruskal–Wallis test, and pairwise comparisons were analyzed with the post‐hoc Dunn test. Data are given as mean ± standard deviation.

In this study, it was found that ovariectomy led to an increase in anxiety and depression‐like behavior in rats. The forced swimming test indicated depression‐like behavior, as evidenced by an increase in immobilization time and reduced climbing time (Figure 2a,b) (Yankelevitch‐Yahav et al., 2015). On the other hand, in the open field test, an increase in the latency time in the center, a decrease in the total number of passed frames and a decrease in the number of rearings indicate anxiety‐like behavior (Figure 3a–c) (Seibenhener & Wooten, 2015). There was a significant decrease in the immobility time of ovariectomized rats administered AT1RB compared to rats treated with ovx only (*p* = 0.045). There was a significant decrease in climbing times in the OVX group compared to the control and AT1RB groups (*p* = 0.038), and the AT1RB + OVX group showed a significant increase compared to the OVX group (*p* = 0.025). The total number of frames passed by the subjects showed a statistically significant decrease in the OVX group compared to the control group (*p* = 0.046) and AT1RB group (*p* = 0.003). A significant increase was seen in the AT1RB + OVX group compared to the OVX group. There was a significant increase in latency time in the OVX group compared to the control group (*p* = 0.032), and the AT1RB + OVX group showed a significant decrease compared to the OVX group (*p* = 0.019). The number of subjects standing up and rearing up on their hind limbs showed a statistically significant decrease in the OVX group compared to the control group (*p* = 0.031) and the AT1RB group (*p* = 0.007), and the increase in the values was found to be significant when the AT1RB + OVX group was compared with the OVX group (*p* = 0.019). These results show that menopause increases depression and anxiety‐like behaviors.

### 3.3 AT1RB reduced oxidative stress increased by Ovariectomy in the hippocampus

**FIGURE 4:**
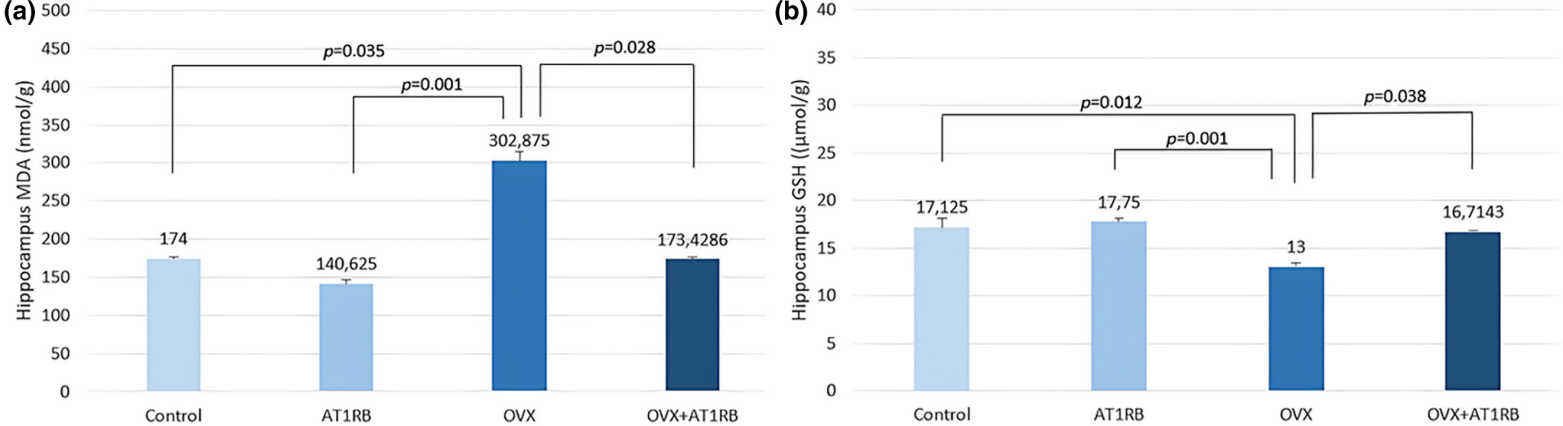
(a, b) Oxidative stress biomarkers in the hippocampus. Oxidative stress increased in the hippocampus of rats showing depression and anxiety‐like behavior. Valsartan treatment maintained oxidative stress at control group levels. Intergroup comparisons were analyzed with the Kruskal–Wallis test, and pairwise comparisons were analyzed with the post‐hoc Dunn test. Data are given as mean ± standard deviation. MDA; malondialdehyde, GSH; reduced glutathione.

Oxidative stress plays an important role in the emergence of depression‐like behavior (Abd El‐Fattah et al., 2018). Estrogens can prevent the production of ROS and lipid peroxidation and change the production of antioxidant enzymes (Behling et al., 2015). MDA values measured in the hippocampus increased significantly in the OVX group compared to the control group (*p* = 0.035) and AT1RB group (*p* = 0.001). The AT1RB + OVX group showed a statistically significant decrease compared to the OVX group (*p* = 0.028). GSH values in the OVX group decreased statistically significantly compared to the control group (*p* = 0.012) and AT1RB group (*p* = 0.001). The AT1RB + OVX group increased significantly compared to the OVX group (*p* = 0.038). These results show that depression due to experimental menopause model increases oxidative stress in the hippocampus region of the brain and that valsartan treatment reduces oxidative stress. (Figure 4a,b). These results obtained in our study are similar to the study conducted by Behling et al. (2015). These researchers also found that oxidative stress increased in the hippocampus after ovariectomy. Additionally, increased oxidative stress in the brains of depressed patients has been shown postmortem (Gawryluk et al., 2011).

### 3.4 Effect of AT1RB and Ovariectomy on BDNF, NLRP3, IL‐1β levels in hippocampus

**FIGURE 5:**
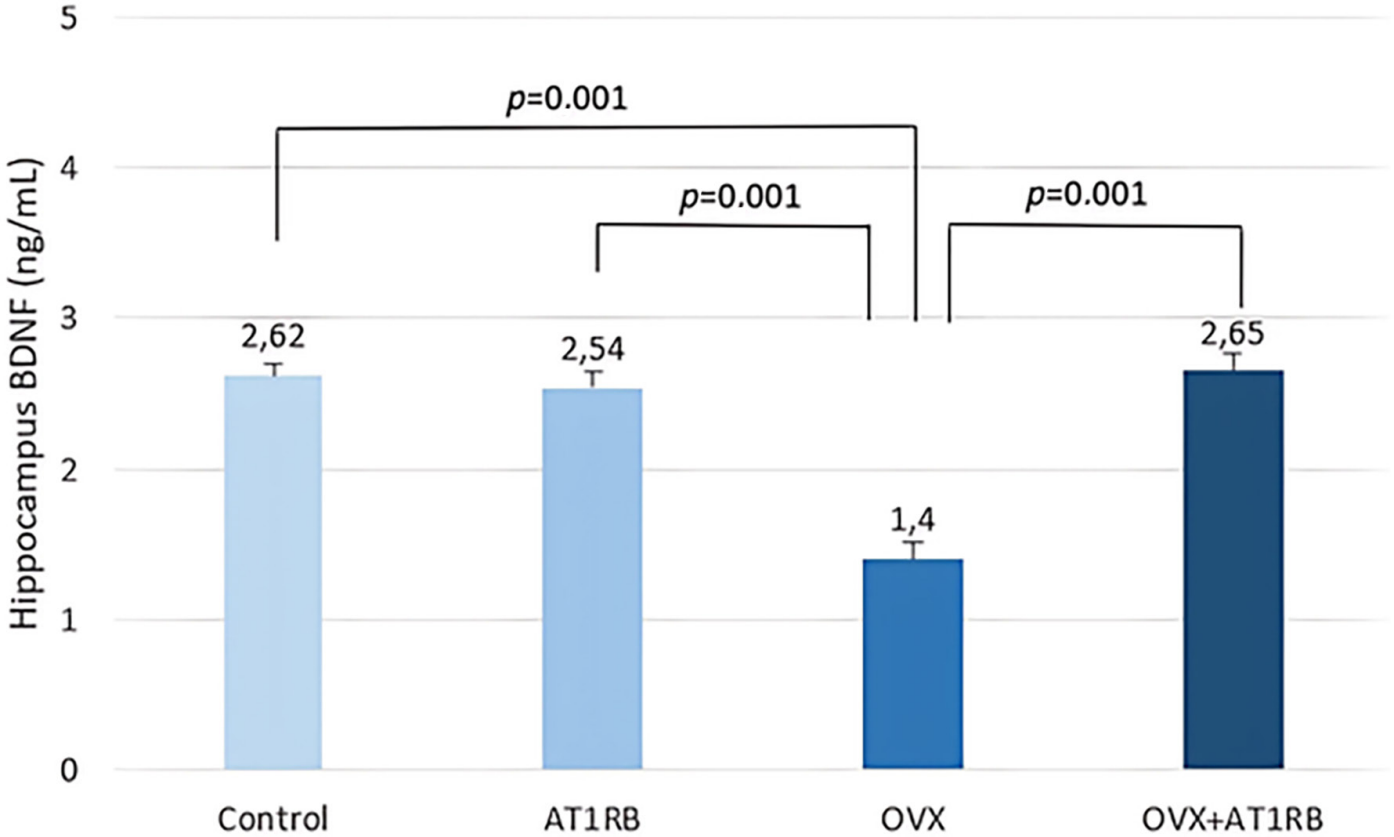
Comparison of hippocampus BDNF levels between groups. BDNF levels decreased in the hippocampus of rats showing depression and anxiety‐like behavior. Valsartan treatment maintained BDNF levels at control group levels. Intergroup comparisons were analyzed with the Kruskal–Wallis test, and pairwise comparisons were analyzed with the post‐hoc Dunn test. Data are given as mean ± standard deviation. BDNF; brain derived neurotrophic factor.

**FIGURE 6:**
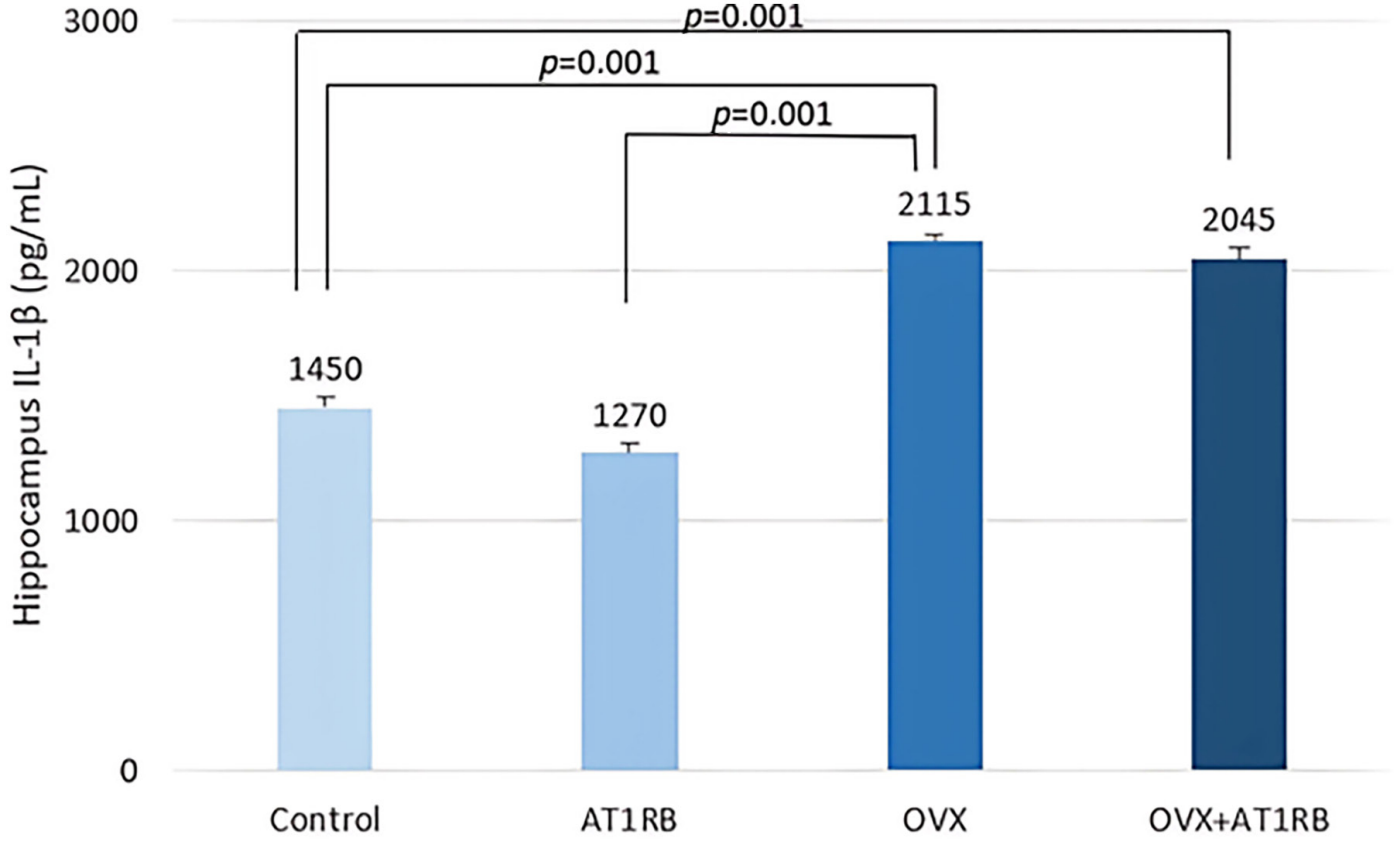
Comparison of hippocampus IL‐1β levels between groups. Ovariectomy increased IL‐1β levels in the hippocampus of the rats, and valsartan was not effective in maintaining IL‐1β levels. Intergroup comparisons were analyzed with the Kruskal–Wallis test, and pairwise comparisons were analyzed with the post‐hoc Dunn test. Data are given as mean ± standard deviation. IL‐1β; Interleukin 1 beta.

**FIGURE 7:**
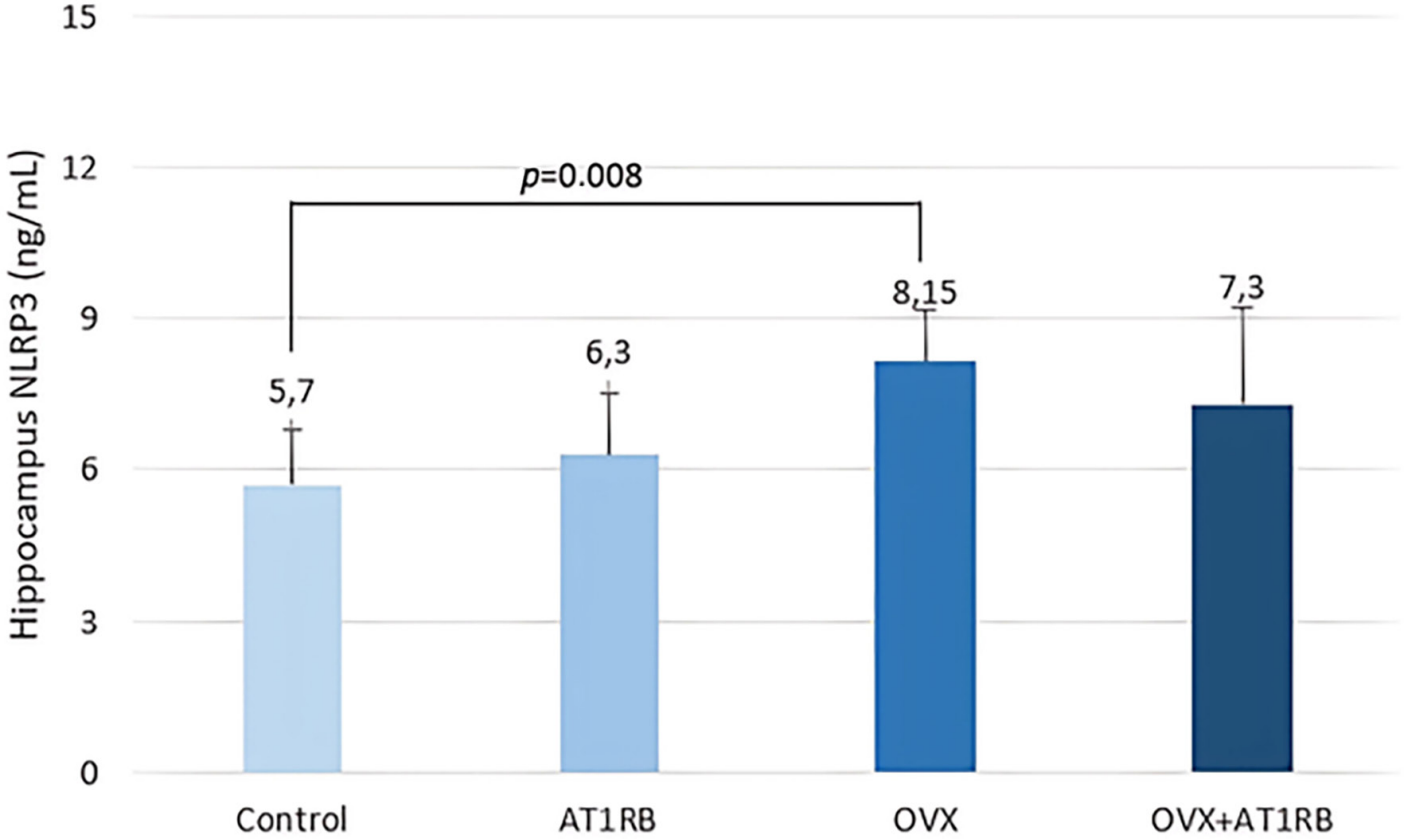
Comparison of hippocampus NLRP3 levels between groups. Ovariectomy increased NLRP3 levels in the hippocampus of rats, and valsartan was not effective in preserving NLRP3 levels. Intergroup comparisons were analyzed with the Kruskal–Wallis test, and pairwise comparisons were analyzed with the post‐hoc Dunn test. Data are given as mean ± standard deviation. NLRP3; NLR family pyrin domain containing 3.

It was found that the decrease in anxiety and depression behaviors after valsartan treatment in rats exposed to chronic stress was accompanied by an increase in BDNF production (Ping et al., 2014). In addition, decreased BDNF levels, especially in the hippocampal area, in ovariectomized female rats are thought to be associated with depression‐like behavior (Lu et al., 2014). In our study, BDNF level in the hippocampus decreased with ovariectomy and BDNF level was preserved with valsartan treatment (Figure 5). Additionally, in our study, we found that ovariectomy increased IL‐1β (Figure 6) and NLRP3 (Figure 7). levels in the hippocampus, while valsartan administration did not affect their levels. It appears that increased production of IL‐1β and NLRP3 in the hippocampus may be effective in the development of increased depression‐like behaviors after ovariectomy.

## 4 DISCUSSION

Some clinical studies show that the use of AT1R blocks as antihypertensives reduces the incidence of dementia development, but antihypertensive activity may also play a role in this protective effect (Forette et al., 1998). In a recent study, it has been reported that the neuroprotective activity of AT1RBs occurs mostly independently of the antihypertensive effect (Campos et al., 2020). In addition, Li et al. showed that AT1R was protective against postoperative cognitive dysfunctions without any change in blood pressure (Li et al., 2010). In our study, valsartan, an AT1RB, effectively reduced the neurobehavioral changes that developed with ovariectomy without any change in blood pressure. These findings are similar to the results of Glenda et al. study in that AT1RBs showed a protective effect on behavioral changes without changes in blood pressure (Campos et al., 2020).

No specific pathophysiological process has been associated with neuronal morphological changes due to depression. Volumetric decreases in the prefrontal cortex (PFC) and hippocampus in depressive disorders have been reported from brain imaging studies (Campbell et al., 2004). One of the most likely causes for these neuronal changes is high oxidative stress due to increased production of free radicals. A growing body of literature, not only in humans but also in preclinical findings in animal models, supports the oxidative stress hypothesis of depressive disorder. The decrease in GSH level and high MDA level in the hippocampus observed in this study may reflect the inability of the hippocampus to eliminate ROS triggered by ovariectomy or the inactivation of antioxidant enzymes due to an excessive increase in ROS. In our study, menopause increased depression and anxiety‐like behavior, and valsartan treatment prevented neurobehavioral disorders by reducing oxidative stress.

It is known that physiological and psychological stresses inhibit the proliferation of precursor cells in the hippocampal subgranular area and the formation of hippocampal granular neurons. The use of antidepressants can counteract this effect. This effect is associated with an increase in BDNF and TrkB receptor production (Lu et al., 2014). It is important to extend these findings and examine the behavioral effects of valsartan in a model of depression and anxiety in ovariectomized rats. It is observed that the antidepressant and anxiolytic effects of valsartan are related to the increased BDNF level in the hippocampus. The renin‐angiotensin‐aldosterone system can affect the amount of BDNF and TrkB receptors in the brain. In an experimental model of cerebral ischemia, candesartan, an AT1RB, was found to reduce infarct volume and neurological damage while also increasing TrkB receptor mRNA production (Krikov et al., 2008). Brain AT1 receptors play an important role in the physiological control of brain inflammation, cerebrovascular function and stress. The results of studies conducted with AT1R blockers show that uncontrolled excessive activation of AT1 receptors is characterized by a pathological response to inflammation and stress (Rao et al., 2023). AT1 blockers are effective in reducing brain inflammation after systemic inflammation caused by lipopolysaccharide, cerebrovascular inflammation caused by hypertension, cerebral ischemia and brain inflammatory response after traumatic brain injury (Benicky et al., 2011; Sironi et al., 2004; Timaru‐Kast et al., 2012; Zhou et al., 2005). The use of systemic AT1R blockers antagonizes peripheral AT1 receptors found in vascular endothelial cells, macrophages and adrenal glands, resulting in a decrease in proinflammatory cytokine production. In addition, stimulation of AT1 receptors causes an increase in intracellular inflammatory events and oxidative stress, along with the activation of nuclear factor kappa B (NF‐κB), which is a transcription factor involved in the production of cytokines (Saavedra, 2012). In addition to these effects, AT1RB can also prevent inflammation in brain tissue through the activation of microglial and astrocytes. It has been shown that AT1RBs cause the inflammatory response in brain tissue caused by LPS by microglial activation and inhibition of IL‐1β production (Saavedra et al., 2011). In our study, we found that ovariectomy increased IL‐1β levels in the hippocampus, and administration of valsartan did not affect IL‐1β levels in the hippocampus. It appears that the increase in IL‐1β production in the hippocampus may be effective in the development of increased depression‐like behavior after ovariectomy. During the transition to menopause, an increase in susceptibility to depression and stress sensitivity is observed in women. Estrogens affect the function of brain areas related to mood and emotional behavior. Various studies have revealed a close relationship between low estrogen levels and emotional state‐related diseases (Fedotova et al., 2016; Xu et al., 2015a). Recently, it has been reported that neuroinflammation plays a central role in the development of depressive symptoms. Various studies have shown that neuroinflammation in depression models is characterized by the release of toll‐like receptor 4 (TLR‐4), NF‐κB, and proinflammatory cytokines (Gárate et al., 2011; Liu et al., 2021; Zhou et al., 2020). Recently, it has been reported that inflammasomes, especially NLRP3, play an important role in the development of the inflammatory response of the central nervous system. It is also suggested that NLRP3 plays a role in the pathogenesis of depression. NLRP3 activation occurs in major depressive patients and experimental studies have shown that it is effective in the formation of depression‐like behavior. It is reported that neuroinflammation is involved in the pathophysiology of depression caused by estrogen deficiency. It was determined that IL‐1β levels along with NLRP3 increased in the hippocampus of female rats after ovariectomy. IL‐1β is a proinflammatory cytokine that plays a fundamental role in the emergence of depression‐like behavior. While administration of IL‐1β to the intracerebroventricular area caused depression‐like behavior, it was observed that depression behavior did not occur in mice with IL‐1β receptor deficiency. Wang et al. report that increased IL‐1β is effective in the formation of depression‐like behavior in animals without ovaries (Wang et al., 2016). In this study, an increase in the level of NLRP3 was detected in the hippocampal area after ovariectomy. Considering this information, our study observes that the physiopathology of depression‐like behavior after ovariectomy includes an increase in NLRP3 and IL‐1β levels and a decrease in BDNF levels in the hippocampal area. Depression‐like behavior has been shown to decrease with AT1RB. It seems that this effect is caused by preserving BDNF levels rather than reducing pro‐inflammatory processes in the hippocampal area.

## 5 CONCLUSIONS

In our study, depression and anxiety‐like behaviors were observed to increase in ovariectomized rats. These behavioral disorders caused oxidative stress damage in the hippocampus and changes in NLRP3, IL‐1β and BDNF levels. Treatment with valsartan, an angiotensin II type I receptor blocker, reduced depression and anxiety‐like behavioral disorders and protected oxidative stress and BDNF levels. However, valsartan did not show any effect on NLRP3 and IL‐1β levels. As a result, it has been shown for the first time that the antidepressive and antianxiety properties of the AT1 receptor blocker valsartan are mediated by oxidative stress levels and BDNF in the hippocampus. Considering the high comorbidity between depression and cardiovascular disorders, our results suggest that valsartan may be a potential therapeutic option or even a new class of antidepressant drugs.

## 6 LIMITATIONS

The sample size used in the study may have been limited. Larger sample sizes may increase the generalizability of results and allow for more robust statistical analysis. The short‐term effects of valsartan treatment were examined. Longer‐term studies are required to evaluate long‐term treatment effects. It was performed only on female Wistar albino rats. Studies in different genders and animal models can increase the generalizability of the results and reveal the effects of gender differences. The focus was on NLRP3, IL‐1β, BDNF, and oxidative stress markers. However, to fully understand the effects of AT1RB on menopause‐induced depression and anxiety, other molecular and cellular mechanisms need to be examined. It may not always be possible to generalize findings obtained in animal models to humans. Therefore, human studies are required to evaluate the clinical relevance of the findings. These limitations should be considered when interpreting the findings of our study and guiding future research. Larger, longer‐term studies are necessary to confirm and extend these findings.

## AUTHOR CONTRIBUTIONS

*Concept*: H.S.Ö., S.E. *Design*: H.S.Ö., S.E., İ.T. *Data Collection or Processing*: H.S.Ö., S.E., İ.T., M.E. *Analysis or Interpretation*: İ.T., M.E. *Literature search*: İ.T., M.E. *Writing*: S.E., H.S.Ö. *Approved*: S.E., H.S.Ö., İ.T., M.E.

## FUNDING INFORMATION

This study received no financial support.

## CONFLICT OF INTEREST STATEMENT

The authors comply with the conflict of interest disclosure guidelines set by the International Committee of Medical Journal Editors (ICMJE). None of the authors involved in this study have any conflict of interest through financial support, sponsorship, funding, paid consultancy, shareholding, patent licensing, expertise, training, or other financial relationships directly or indirectly related to the content of this article.

## ETHICS STATEMENT

This study was carried out following the terms of the Zonguldak Bülent Ecevit University Animal Experiments Local Ethics Committee (protocol number: 2018‐08‐05/04).

## Data Availability

The authors declare that the data supporting the findings of this study are available within the paper and its Supplementary Information files. Any remaining data that support the results of the study will be availablefrom the corresponding authors upon reasonable request.
